# Red pitaya juice supplementation ameliorates energy balance homeostasis by modulating obesity-related genes in high-carbohydrate, high-fat diet-induced metabolic syndrome rats

**DOI:** 10.1186/s12906-016-1200-3

**Published:** 2016-07-26

**Authors:** Nurul Shazini Ramli, Patimah Ismail, Asmah Rahmat

**Affiliations:** 1 Department of Food Science, Universiti Putra Malaysia, Serdang, Selangor 43400 Malaysia; 2 Department of Biomedical Science, Universiti Putra Malaysia, Serdang, 43400 Malaysia; 3 Department of Nutrition and Dietetics, Universiti Putra Malaysia, Serdang, 43400 Malaysia

**Keywords:** Red pitaya juice, Obesity-related genes, Metabolic syndrome

## Abstract

**Background:**

Red pitaya (*Hylocereus polyrhizus*) or known as *buah naga merah* in Malay belongs to the cactus family, *Cactaceae*. Red pitaya has been shown to give protection against liver damage and may reduce the stiffness of the heart. Besides, the beneficial effects of red pitaya against obesity have been reported; however, the mechanism of this protection is not clear. Therefore, in the present study, we have investigated the red pitaya-targeted genes in obesity using high-carbohydrate, high-fat diet-induced metabolic syndrome rat model.

**Methods:**

A total of four groups were tested: corn-starch (CS), corn-starch + red pitaya juice (CRP), high-carbohydrate, high-fat (HCHF) and high-carbohydrate, high-fat + red pitaya juice (HRP). The intervention with 5 % red pitaya juice was continued for 8 weeks after 8 weeks initiation of the diet. Retroperitoneal, epididymal and omental fat pads were collected and weighed. Plasma concentration of IL-6 and TNF-α were measured using commercial kits. Gene expression analysis was conducted using RNA extracted from liver samples. A total of eighty-four genes related to obesity were analyzed using PCR array.

**Results:**

The rats fed HCHF-diet for 16 weeks increased body weight, developed excess abdominal fat deposition and down-regulated the expression level of IL-1α, IL-1r1, and Cntfr as compared to the control group. Supplementation of red pitaya juice for 8 weeks increased omental and epididymal fat but no change in retroperitoneal fat was observed. Red pitaya juice reversed the changes in energy balance homeostasis in liver tissues by regulation of the expression levels of Pomc and Insr. The increased protein expression levels of IL-6 and TNF-α in HCHF group and red pitaya treated rats confirmed the results of gene expression.

**Conclusion:**

Collectively, this study revealed the usefulness of this diet-induced rat model and the beneficial effects of red pitaya on energy balance homeostasis by modulating the anorectic, orexigenic and energy expenditure related genes.

## Background

The increasing incidence of obesity worldwide could result from complex interactions between environmental, genetic [1] and psychosocial factors [2]. The social-environmental influences such as reduced physical activity, increased sedentary behavior and increased accessibility of high-fat and energy-dense foods facilitate the obesity pandemic by disrupting the body’s energy balance. Energy balance is defined as the difference between energy intake and energy expenditure including basal metabolism, physical activity and adaptive thermogenesis [2]. A state of positive energy balance will occur when energy intake exceeds energy expenditure, leading to increased storage of energy as adipose tissue. Genetic factors may also influence body weight by affecting one or more component (s) of energy balance [3].

The physiological control system for energy intake and body weight is complex and involves extensive changes of gene expression throughout the body. It is postulated that the central nervous system controls energy balance through several mechanisms. These include it influence on feeding and physical activity behavior, the regulation of the autonomic nervous system activities including metabolism, and changes in key hormones such as leptin, ghrelin, insulin, growth hormone, sex steroids, cortisol and thyroid hormones [1, 4–6]. Therefore, it is critical to understand the gut-brain interaction underlying the appetite and feeding regulation of energy balance to develop new pharmacogenetical strategy for obesity studies.

Red pitaya is a cactus fruit originating from Mexico. In Malaysia, this fruit is known as red dragon fruit or ‘*buah naga merah’*, possibly due to the scaly structure of the peel. The beneficial effects of red pitaya reported in laboratory animal studies could be due to its ability to increase antioxidant capacity and suppress oxidative stress damage [7–9]. Our previous study indicated that red pitaya supplementation ameliorated liver and cardiovascular damage induced by high-carbohydrate, high-fat feeding [10]. However, the effects of red pitaya supplementation on obesity along with its related mechanism not clear. Red pitaya supplementation increases energy intake without increasing body weight [10], so it is hypothesized that red pitaya may stimulate anorectic genes or down-regulate orexigenic genes to increase energy expenditure. Therefore, the present work determined the changes in anorectic and orexigenic genes as well as the inflammatory pathway in a rat model of diet-induced obesity following supplementation with red pitaya.

## Methods

### Preparation of diet

Red pitaya was obtained from Queensland Australia. The identification of the fruit has been done by a botanist from Biodiversity Unit, Institute of Biosciences, Universiti Putra Malaysia. The voucher number is SK-2440/14. The fruits were then cleaned, and the fruit pulp was squeezed using juice maker. Sample preparation was conducted in reduced light condition in order to minimize the pigment loss [10].

### Animals and diet

The experimental protocols have been described in our previous publication [10]. In brief, the experimental groups consisted of 48 male Wistar rats (aged 8–9 weeks; weight 337 ± 5 g) supplied by and individually housed at The University of Southern Queensland animal house. All experimental groups were housed in a temperature-controlled, 12 hour light–dark cycle environment with *ad libitum* access to water and the group specific diet. Daily body weight, feed and water measurements were taken to monitor the day-to-day health of the rats and the results have been reported in our previous publication [10]. The rats were randomly divided into four groups based on their diet: corn starch (CS; *n* = 12); corn starch + red pitaya juice (CRP; 5 % in the diet; *n* = 12); high-carbohydrate, high-fat (HCHF; *n* = 12); High-carbohydrate, high-fat + red pitaya juice (HRP; *n* = 12). Fructose (25 %) was added as drinking water for all high-carbohydrate, high-fat fed rats, while corn starch group was given normal water. The detailed macro- and micro-nutrient composition of the CS and HCHF diets are reported in previous publications [11, 12]. Red pitaya juice was administered for 8 weeks starting from 8 weeks after the initiation of the CS or HCHF diet. All the experimental protocols were approved by the Animal Experimentation Ethics Committee of The University of Southern Queensland under the guidelines of the National Health and Medical Research Council of Australia.

### Organ weights

Terminal anaesthesia was induced via intraperitoneal injection of pentobarbitone sodium (Lethabarb, 100 mg/kg). Heparin (Sigma-Aldrich Australia) was administered (100 IU) through the right femoral vein and blood (about 5 ml) was taken from the abdominal aorta. Immediately after the rats were killed, the abdominal fat mass as the retroperitoneal, epididymal and omental fat pads were collected. The organ weights were normalized to the tibial length at the time of their removal (in mg/mm).

### Visceral adiposity index

Visceral adiposity index (%) was calculated as: ([retroperitoneal fat (g) + omental fat (g) + epididymal fat (g)]/[body weight (g)]) × 100 and expressed as adiposity percent [12].

### Inflammatory markers

Plasma concentrations of interleukin 6 (IL-6) and TNF-alpha were quantified based on manufacturer’s guidelines using commercially available ELISA kits. Plasma C-reactive protein (CRP) was estimated using a commercial kit according to the manufacturer-provided standards and protocol using a Roche/Hitachi cobas c system.

### Isolation of total RNA

During terminal experiments, liver samples were collected and immediately stored at −80 °C freezer. Total RNA from corn starch (CS), corn starch + red pitaya (CRP), high-carbohydrate, high-fat (HCHF) and high-carbohydrate, high-fat + red pitaya (HRP) groups was isolated from liver samples using RNeasy® plus mini kit (Qiagen, Valencia, CA) according to manufacturer’s protocol. Three replicates were analyzed for each group (CS, CRP, HCHF and HRP). Basically, 25 mg liver samples were excised and immediately homogenized in Buffer RLT plus. The genomic DNA was removed and purified from the lysate liver sample. Finally, the RNeasy spin column was placed into a new 2 mL collection tube and centrifuged at 12000 rpm for 1 minute. The 2 mL collection tube was then discarded. The RNA bound at the RNeasy spin column was eluted in a new 1.5 mL collection tube with 40 μL RNase-free water and centrifuged at 12000 rpm for 1 minute. The concentration of extracted RNA from the samples was determined by measuring at 260 nm using a Nano-Drop 1000 Spectrophotometer (Thermo Scientific, Waltham, MA). In brief, 1 μL of RNase-free water was added to pedestal for blank sample. Then, 1 μL of RNA sample was added. The integrity of extracted RNA sample was measured using Bioanalyzer (Agilent’s RNA 6000 Nano kit).

### cDNA synthesis for real-time Reverse Transcription- (RT) PCR

cDNA was prepared using RT^2^ profiler PCR array first strand kit based on manufacturer’s instructions. Briefly, genomic DNA elimination mix was prepared by mixing RNA (volume varies based on the concentration obtained), 2 μL of gDNA elimination buffer and RNase-free water to form a total volume of 10 μL. The mixture was mixed by gently pipetting and the genomic DNA elimination mix was then incubated for 5 minutes at 42 °C. It was immediately placed on ice for at least 1 minute. The reverse-transcription mix was prepared according to manufacturer’s guidelines. Ten μL of reverse-transcription mix was added to tube containing 10 μL genomic DNA elimination mix, and incubated at 42 °C for exactly 15 minutes. The reaction was stopped by incubating at 95 °C for 5 minutes to inactivate the reverse transcriptase. The 20 μL of cDNA synthesis reaction mixture was mixed with 91 μL of RNase-free water making a total 111 μL of reaction mixture. The reaction mixture was placed on ice until further use.

### Array-based SYBR® Green RT-PCR

The constitutive gene expression profiling was conducted using to RT^2^ profiler PCR array related to obesity signal transduction according to manufacturer’s protocols. The gene array profiled the expression of 84 genes including orexigenic genes, anorectic genes, and related to energy expenditure (Table 1, PARN-017Z-12, RT^2^ Profiler™ PCR Array Rat Obesity). The array included the controls for human genomic DNA contamination, reverse-transcription, positive PCR control, and 5 housekeeping genes to normalize the relative gene expression for analysis of data. The five housekeeping genes were ribosomal protein large P1 (Rp1p1), hypoxanthine phosphoribosyltransferase 1 (Hprt1), ribosomal protein L13A (Rp113a), lactate dehydrogenase A (Ldha), and β-actin (Actb). The PCR components mix were prepared with 1150 μL of 2 x RT^2^ SYBR Green ROX FAST mastermix, 102 μL of cDNA synthesis reaction, and 1048 μL of RNase-free water combined making a total volume of 2300 μL. Then, the RT^2^ profiler PCR array was removed from its sealed bag, and the array was slid into the Rotor-Disc 100 Loading Block using the tab position A1 and the tube guide holes. Twenty μL of PCR component mix was pipetted into each well of the RT^2^ profiler PCR array. For each sample, the RT^2^ profiler PCR array was conducted in triplicate.

**Table 1:** The symbol and description of genes in the PCR array

Position	Symbol	Description	Gene Name
A01	Adcyap1	Adenylate cyclase activating polypeptide 1	Pacap
A02	Adcyap1r1	Adenylate cyclase activating polypeptide 1 receptor 1	PACAP-R1A, PACAPR1, PACAPR1A
A03	Adipoq	Adiponectin, C1Q and collagen domain containing	Acdc, Acrp30
A04	Adipor1	Adiponectin receptor 1	–
A05	Adipor2	Adiponectin receptor 2	–
A06	Adra2b	Adrenergic, alpha-2B-, receptor	–
A07	Adrb1	Adrenergic, beta-1-, receptor	B1AR,RATB1AR
A08	Agrp	Agouti related protein homolog (mouse)	–
A09	Apoa4	Apolipoprotein A-IV	Apo-AIV, ApoA IV, apoAIV
A10	Atrn	Attractin	–
A11	Bdnf	Brain-derived neurotrophic factor	MGC105254
A12	Brs3	Bombesin-like receptor 3	–
B01	C3	Complement component 3	–
B02	Calca	Calcitonin-related polypeptide alpha	CAL6, CGRP, Cal1, Calc, RATCAL6, calcitonin
B03	Calcr	Calcitonin receptor	–
B04	Cartpt	CART prepropeptide	Cart
B05	Cck	Cholecystokinin	–
B06	Cckar	Cholecystokinin A receptor	Cck-ar
B07	Clps	Colipase, pancreatic	COLQ
B08	Cnr1	Cannabinoid receptor 1 (brain)	SKR6R
B09	Cntf	Ciliary neurotrophic factor	–
B10	Cntfr	Ciliary neurotrophic factor receptor	–
B11	Crh	Corticotropin releasing hormone	CRF
B12	Crhr1	Corticotropin releasing hormone receptor 1	–
C01	Drd1a	Dopamine receptor D1A	D1a, Drd-1, Drd1
C02	Drd2	Dopamine receptor D2	–
C03	Gal	Galanin prepropeptide	Galn
C04	Galr1	Galanin receptor 1	Galnr1
C05	Gcg	Glucagon	GLP-1
C06	Gcgr	Glucagon receptor	MGC93090
C07	Gh1	Growth hormone 1	Gh,RNGHGP
C08	Ghr	Growth hormone receptor	GHR, BP, MGC12496, MGC156665
C09	Ghrl	Ghrelin/obestatin prepropeptide	–
C10	Ghsr	Growth hormone secretagogue receptor	–
C11	Glp1r	Glucagon-like peptide 1 receptor	Glip, RATGL1RCP
C12	Grp	Prolactin releasing hormone receptor	Gpr10, Uhr-1
D01	Grpr	Melanin-concentrating hormone receptor 1	Gpr24, Mch-1r, Slc1
D02	HcRt	Hypocretin	orexin-A
D03	Hcrtr1	Hypocretin (orexin) receptor 1	Hctr1
D04	Hrh1	Histamine receptor H 1	Hisr
D05	Htr2c	5-hydroxytryptamine (serotonin) receptor 2C 1C	5-HT2C, 5-HTR2C, 5HT-
D06	Iapp	Islet amyloid polypeptide	–
D07	IL-1α	Interleukin 1 alpha	IL-1 alpha
D08	IL-1b	Interleukin 1 beta	–
D09	IL-1r1	Interleukin 1 receptor, type I	–
D10	IL-6	Interleukin 6	ILg6, Ifnb2
D11	IL-6r	Interleukin 6 receptor	IL6R1, Il6ra, Il6r
D12	Ins1	Insulin 1	–
E01	Ins2	Insulin 2	–
E02	Insr	Insulin receptor	–
E03	Lep	Leptin	OB, obese
E04	Lepr	Leptin receptor	Fa
E05	Mc3r	Melanocortin 3 receptor	MC3-R
E06	Mchr1	Melanin-concentrating hormone receptor 1	Gpr24, Mch-1r, Slc1
E07	Nmb	Neuromedin	B RGD1562710
E08	Nmbr	Neuromedin B receptor	NMB-R
E09	Nmu	Neuromedin U	–
E10	Nmur1	Neuromedin U receptor 1	Gpr66
E11	Npy	Neuropeptide Y	NPY02, RATNPY, RATNPY02
E12	Npy1r	Neuropeptide Y receptor Y1	MGC109393, NPY-1
F01	Nr3c1	Nuclear receptor subfamily 3, group C, member 1	GR, Gcr, Grl
F02	Ntrk1	Neurotrophic tyrosine kinase, receptor, type 1	Trk
F03	Nts	Neurotensin	–
F04	Ntsr1	Neurotensin receptor 1	Ntsr
F05	Oprk1	Opioid receptor, kappa 1	–
F06	Oprm1	Opioid receptor, mu 1	MORA, Oprm, Oprrm1
F07	Pomc	Proopiomelanocortin	Pomc1, Pomc2
F08	Ppara	Peroxisome proliferator activated receptor alpha	PPAR
F09	Pparg	Peroxisome proliferator-activated receptor gamma	–
F10	Ppargc1a	Peroxisome proliferator-activated receptor gamma, coactivator 1 alpha	Ppargc1
F11	Prlhr	Prolactin Releasing Hormone Receptor	Prlhr
F12	Ptpn1	Protein tyrosine phosphatase, non-receptor type 1	MGC93562, Ptp
G01	Pyy	Peptide YY (mapped)	GHYY, RATGHYY, Yy, peptide-YY
G02	Ramp3	Receptor (G protein-coupled) activity modifying protein 3	–
G03	Sigmar1	Sigma non-opioid intracellular receptor 1	Oprs1
G04	Sort1	Sortilin 1	Nt3, Nts3
G05	Sst	Somatostatin	SS-14, SS-28, Smst
G06	Sstr1	Somatostatin receptor 1	Gpcrrna
G07	Thrb	Thyroid hormone receptor beta	C-erba-beta, ERBA2, Nr1a2, RATT3REC, T3rec, TRbeta
G08	Tnf	Tumor necrosis factor (TNF superfamily, member 2)	MGC124630, RATTNF, TNF-alpha, Tnfa
G09	Trh	Thyrotropin releasing hormone	THR, TRH01
G10	Trhr	Thyrotropin releasing hormone receptor	–
G11	Ucn	Urocortin	–
G12	Ucp1	Uncoupling protein 1 (mitochondrial, proton carrier)	MGC108736, Ucp, Ucpa, Uncp
H01	Actb	Actin, beta	Actx
H02	B2m	beta-2-microglobulin	-
H03	Hprt1	Hypoxanthine phosphoribosyltransferase 1	Hgprtase, Hprt, MGC112554
H04	Ldha	Lactate dehydrogenase A	Ldh1
H05	Rplp1	Ribosomal protein, large, P1	MGC72935
H06	RGDC	Rat Genomic DNA Contamination	RGDC
H07	RTC	Reverse Transcription Control	RTC
H08	RTC	Reverse Transcription Control	RTC
H09	RTC	Reverse Transcription Control	RTC
H10	PPC	Positive PCR Control	PPC
H11	PPC	Positive PCR Control	PPC
H12	PPC	Positive PCR Control	PPC

(Source: SABioscience, 2014)

Real time PCR was performed using a two-step cycling program on rotor gene real time PCR machine (Qiagen, Valencia, CA): 10 min at 95 °C (cycle 1) followed by 40 cycles of 15 s at 95 °C and 1 min at 60 °C. SYBR green fluorescence was detected and recorded. The threshold cycle (CT) above the background for each reaction was then calculated.

### Statistical analysis

Data for organ weights, and inflammatory markers were analyzed using GraphPad Prism version 5.00 for Windows (San Diego, CA, USA). All data were presented as mean ± SEM. All group data were tested for variance using Bartlett’s test. Variables that were not normally distributed were transformed (using log 10 function) prior to statistical analysis. The effects of diet, treatment and their interactions were tested by two-way analysis of variance. When interaction and/or the main effects were significant, means were compared using Newman-Keuls multiple-comparison *post hoc* test. A nonparametric test, the Kruskal-Wallis test, was performed when transformations did not result in normality or constant variance. The gene expression data was analyzed using RT^2^ Profiler PCR Array Data analysis version 3.5 from SABiosciences website. All data were normalized by one housekeeping gene (endogenous control). The fold change among the groups were obtained from ∆∆CT. ∆CT was defined as the value of subtracting the CT value of endogenous control from the CT value of the target messenger RNA (mRNA). Student’s *t*-test was used to determine the differences in gene expression. The significant values were considered at the level of *p* < 0.05.

## Results

### Abdominal fats and visceral adiposity index

An increase in retroperitoneal adipose tissue was observed in CRP but not in HRP compared to their respective non-treated controls (CS and HCHF groups) (Table 2). On the other hand, HRP group showed significant increase (*p* < 0.05) in epididymal and omental fat deposition compared with CS and HCHF diet-fed rats (Table 2). No changes in visceral adiposity index were seen for red pitaya supplementation in high-carbohydrate, high-fat diet-fed rats (Table 2). On the other hand, CRP rats showed a trend of increasing visceral adiposity index but the differences were not significant (*p* > 0.05) (Table 2).

**Table 2:** Visceral adiposity index, abdominal fat, and plasma inflammatory markers in CS, CRP, HCHF and HRP diet-fed rats

Variable	CS	CRP	HCHF	HRP	*P*-value		
					Diet	Treatment	Interaction
Visceral adiposity index (%)	3.5 ± 0.3^bc^	4.6 ± 0.5^b^	6.6 ± 0.6^a^	7.0 ± 0.5^a^	<0.0001	0.05	0.16
Abdominal fat, mg/mm tibial length							
Retroperitoneal (*n* = 7–8)	118.9 ± 12.9^c^	181.7 ± 15.2^b^	303.7 ± 26.6^a^	357.7 ± 32.6^a^	<0.0001	0.0217	0.0341
Omental (*n* = 7–8)	102.5 ± 7.3^cd^	142.4 ± 12.3^cd^	173.9 ± 44.0^bc^	260.3 ± 27.6^a^	<0.0001	0.0141	0.27
Epididymal (*n* = 7–8)	64.4 ± 7.0^cd^	101.3 ± 11.4^bd^	112.1 ± 34.6^bc^	191.0 ± 13.9^a^	<0.0001	0.0012	0.3
Plasma inflammatory markers							
IL-6 (pg/mL) (*n* = 7–8)	3.40 ± 0.48^a^	3.63 ± 0.27^a^	1.82 ± 0.19^b^	3.91 ± 0.56^a^	0.0004	0.0112	<0.0001
TNF-alpha (pg/mL) (*n* = 7–8)	1.08 ± 0.34^b^	1.20 ± 0.16^b^	1.13 ± 0.20^b^	1.83 ± 0.39^a^	0.0448	0.13	0.7465
CRP, mg/L (*n* = 5–8)	0.26 ± 0.04^b^	0.21 ± 0.004^b^	0.34 ± 0.07^a^	0.13 ± 0.02^b^	0.006	<0.0001	0.6422

Each value is a mean ± S.E.M. Number of repetitive experiments indicated within parenthesis. Means within a row with unlike superscripts letters a, b, c, d differ significantly (*p* < 0.05). CS, corn starch diet; CRP, corn starch + red pitaya juice; HCHF, high-carbohydrate, high-fat diet; HRP, high-carbohydrate, high-fat diet + red pitaya juice

### Plasma inflammatory markers

Results indicated that red pitaya supplementation significantly increased (*p* < 0.05) plasma concentration of TNF-α and IL-6 (Table 2). There was no difference for CRP group for IL-6 concentration in comparison with CS-fed rats (Table 2). C-reactive protein, an acute phase reactant was increased with high-carbohydrate, high-fat feeding compared with corn starch fed control rats (Table 2). Red pitaya juice supplementation decreased C-reactive protein concentration in HRP rats as compared to controls rats (Table 2).

### Gene expression analysis

#### Concentration of extracted RNA

Total RNA was successfully extracted from the liver tissues of HCHF, HRP CS, and CRP. For the best results from the RT^2^ Profiler PCR Array, all RNA samples should exhibit consistent quality and purity. High purity of RNA should have an A_260_/A_280_ reading between 1.8 and 2.0 and an A_260_/A_230_ reading should be greater than 1.7. On top of that, the concentration determined by A_260_ should be greater than 40 μg/mL. In the present study, all the samples fulfill the criteria indicating high purity of RNA.

### Gene expressions alterations

**Fig. 1:**
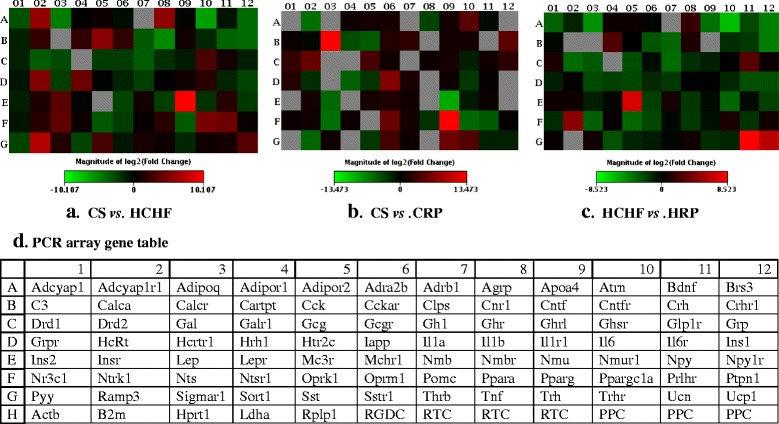
The changes in obesity-related genes among the controls, CRP and HRP groups. The heat map demonstrating fold regulation expression data between CS group and HCHF group (**a**), CS group and CRP group (**b**) and HCHF group and HRP group (**c**). **d** shows representative PCR array gene table

**Fig. 2:**
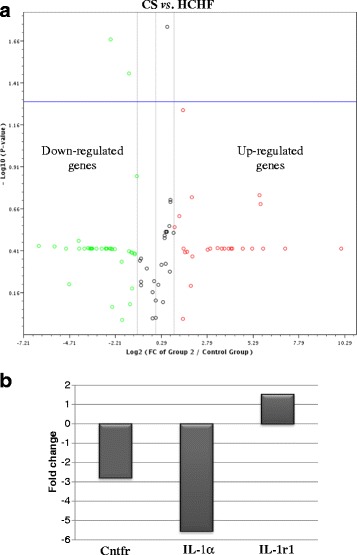
The changes of obesity-related genes from HCHF and CS groups. **a** The volcano plot demonstrating statistical significance versus fold regulation expression data between the HCHF group and the CS group. The blue line indicates *p*-value, set at 0.05. **b** Genes with significant differences (*p* < 0.05) between the two groups are presented in the histogram. CS, corn starch diet; HCHF, high-carbohydrate, high-fat diet; Cntfr, ciliary neurotrophic factor receptor; IL-1 α, Interleukin 1 alpha; IL-1r1, Interleukin 1 receptor, type I

**Fig. 3:**
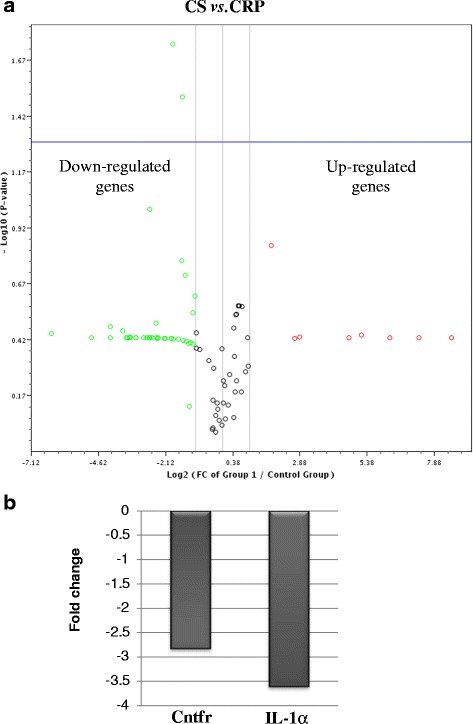
The changes of obesity-related genes from CS and CRP groups. **a** The volcano plot demonstrating statistical significance versus fold regulation expression data between the CS group and the CRP group. The blue line indicates *p*-value, set at 0.05. **b** Genes with significant differences (*p* < 0.05) between the two groups are presented in the histogram. CS, corn starch diet; CRP, corn starch + red pitaya juice; Cntfr, ciliary neurotrophic factor receptor; IL-1 α, Interleukin 1 alpha

**Fig. 4:**
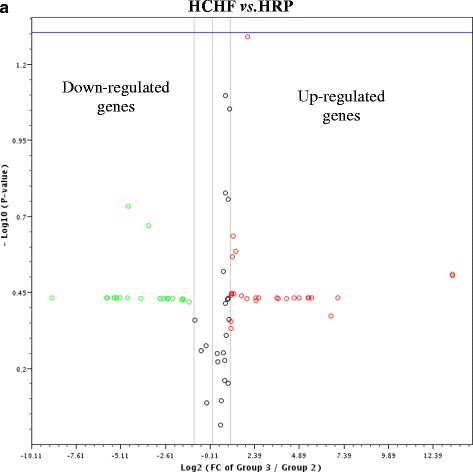
The changes of obesity-related genes from HCHF and HRP groups. **a** The volcano plot demonstrating statistical significance versus fold regulation expression data between the HCHF group and the HRP group. The blue line indicates *p*-value, set at 0.05. None of the genes for this group is significantly different at *p* < 0.05. HCHF, high-carbohydrate, high-fat diet; HRP, high-carbohydrate, high-fat diet + red pitaya juice

The triplicate samples from each group produced reproducible results. Data analysis revealed that 14 genes (as measured by CT value) were detected while the remaining 71 genes were not detected and/or the genes were detected but the variation between the test sample and control is too great and thus the fold change results has to be validated further. Figure 1 shows the fold regulation expression data for all the genes between CS and HCHF groups, CS and CRP groups and HCHF and HRP groups. Table 3 presents the fold regulation of the selected genes from HCHF, HRP, CS and CRP groups that gave reproducible results. In HCHF-fed rats, four genes were down-regulated namely complement component 3 (C3), ciliary neurotrophic factor receptor (Cntfr), glucagon receptor (Gcgr) and interleukin 1 alpha (IL-1α) while three genes were up-regulated which are insulin receptor (Insr), neuropeptides Y (Npy) and Sigma non-opioid intracellular receptor 1 (Sigmar1) and four genes namely Apolipoprotein A-IV (Apoa4), Interleukin 1 receptor, type 1 (IL-1r1), Proopiomelanocortin (Pomc), and Thyroid hormone receptor beta (Thrb) showed no changed as compared to CS group (Table 3). Nevertheless, only the expression level Cntfr, IL-1α and IL-1r1 were significantly different (*p* < 0.05) compared to CS group (Fig. 2). Corn starch-fed supplemented rats (CRP group) significantly decreased (*p* <0.05) the expression of Cntfr and IL-1α (Fig. 3). Supplementation of red pitaya juice for eight weeks resulted in up-regulation of Insr and Pomc in HRP group compared to HCHF group (Table 3). However, the difference showed no statistical significance (*p* > 0.05) (Fig. 4).

**Table 3:** Fold regulations of detected genes from HCHF, HRP, CS and CRP groups

Gene	Fold regulation		
	CS *vs*. HCHF	CS *vs*. CRP	HCHF *vs*. HRP
Adipor1	ND	1.32	1.67
Apoa4	−1.13	−13.15	1.89
C3	−2.47	−1.19	1.43
Cnr1	ND	1.92	ND
Cntfr	−2.79*	−2.82*	ND
Gcgr	−5.17	−1.31	1.74
Ghr	ND	−2.85	ND
IL-1α	−5.55*	−3.60*	ND
IL-1r1	1.53*	1.02	1.67
Insr	2.01	1.37	2.09
Npy	2.79	ND	1.54
Pomc	1.24	−1.20	2.09
Sigmar1	2.43	1.55	1.62
Thrb	1.95	−1.16	1.62

CS, corn starch diet; CRP, corn starch + red pitaya juice; HCHF, high-carbohydrate, high-fat diet; HRP, high-carbohydrate, high-fat diet + red pitaya juice

*indicate significant difference at *p* < 0.05

ND; non-detected genes

## Discussion

The present study evaluated the red pitaya-targeted genes in high-carbohydrate, high-fat diet-induced metabolic syndrome rats using polymerase chain reaction (PCR) array in order to elucidate the molecular mechanism underlying the physiological responses. PCR array combines real-time PCR sensitivity and the ability of microarrays to detect the expression of many genes simultaneously [13]. This new technique of evaluating gene expression allows the expression levels of disease- or pathway-focused genes and provides reliable method, easy-to-use and is highly sensitive [14]. Up to now, quantitative RT-PCR arrays have had limited use to study nutrient-related interventions [15], maternal perinatal under-nutrition [16, 17] and brown adipose tissue metabolism [18], probably because of high cost despite the improved sensitivity.

Analysis from the present study revealed that several obesity-related genes change in HCHF groups, which further confirmed the development of this metabolic syndrome rat model. In obese rats, four genes were down-regulated (C3, Cntfr, Gcgr, IL-1α) while three genes were up-regulated (Insr, Npy and Sigmar1) and five genes (Adipor1, Apoa4, IL-r1, Pomc, Thrb) showed no change compared to CS group. However, among these, only Cntfr, IL-1α and IL-1r1 showed significant changes which might be due to large variations of the expression levels of other genes within the groups. IL-1α and IL-1r1 are classified as anorectic genes. The appetite suppressing effects of these two genes (IL-1α and IL-1r1) might be associated with fever, increased thermogenesis and reduced food intake due to inflammation and injury [19]. Interleukin-1α is cytokine of the interleukin-1 family (IL-1) which is a key mediator of inflammation, located on the long arm of human chromosome 2 [20]. IL-1 family also composed of IL-1β that exerts almost identical biological activities with IL-1α by binding to IL-1 type 1 receptor (IL-1r1). However, IL-1β does not occur in healthy subjects. In contrast, IL-1α is present at constitutive levels in primary cells such as hepatocytes and epithelial cells [21]. IL-1α forms heterodimeric complexes that induce inflammation when bound to the IL-1r1. The negative regulator of inflammation is IL-1 receptor antagonist (IL-1Ra) [18].

García et al. [19] showed that knockout of the gene coding for IL-1r1 resulted in mature-onset obesity due to reduced fat utilization, decreased locomotor activity and reduced leptin sensitivity. Data from our previous study indicated that body weight of HCHF-fed rats were significantly higher than CS-fed rats [10] despite the same expression level of IL-1r1. Possibly, other genes play role in increasing body weight of H-fed rats but not IL-1r1. Furthermore, serum amyloid A protein, an inflammatory marker in atherosclerosis, was reduced despite higher total cholesterol concentrations in IL-1α deficient mice fed with high-fat diet [22]. Similarly, the present study showed the expression level of IL-1α was significantly reduced in line with the decreased inflammatory markers of interleukin-6 in HCHF-fed rats compared to CS group. The relationship between inflammation and obesity is well established [23, 24, 25]. It is interesting to point out that the contradictory results obtained from the present study might be due to increased inflammation in control rats without high-fat diet probably due to enhance glucose intake after meal as reported by Gregerson et al*.* [26]. Importantly, these results explained the condition whereby the appetite suppressing effect of IL-1α was altered resulting in increased food intake, and hence increased body weight of H-fed rats. Recently, Dinarello and Netea [27] postulated that IL-1α deficient mice had reduced aortic lesion size due to the transferred of hematopoietic cells from the bone marrow. However, the aortic lesion was not measured in the present study, so the effect of IL-1α down-regulation on the aortic lesion size was not known.

Cntfr, Gcgr and Insr are anorectic genes, Sigmar1 and Npy are orexigenic genes and C3 is the gene that relate to energy expenditure. Although there were no statistically significant for these genes between the HCHF group and CS group except for Cntfr, it is important to understand the changes of obesity-related genes as a first critical step towards evaluating this metabolic syndrome rat model at the molecular level. The role of C3 in obesity-related metabolic diseases has been recognized as it stimulates the accumulation of triglyceride, promotes the uptake of glucose and reduces the release of free fatty acids [28]. Besides, the up-regulation of C3 impaired energy expenditure and food intake via its action on the central nervous system [29]. It is fascinating that the C3 expression decreases with obesity in the present study given that C3 mRNA expression levels typically positively correlated with obesity. Nevertheless, this finding is in agreement with recent discovery by Gupta et al. [30] whereby they found down-regulation of complement C3 in subcutaneous tissue of obese women.

Cntfr reduces food intake and increase energy expenditure by directly induces the transcription of Pomc genes [31, 32]. Therefore, the absence of this protective factor against high-carbohydrate, high-fat diet resulted in weight gain and obesity. Gcgr is a receptor for glucagon which mediated the process of glycogenolysis, lipolysis, ketogenesis and gluconeogenesis. Following carbohydrate-rich meal, the expression level of Gcgr is significantly decreased [5], which is in agreement with the present study. Furthermore, the increased in Npy and Sigmar1 gene expression further supported this obesity rat model. Npy is part of the hypothalamic melanocortin pathway that regulates the central energy metabolism [33]. Previous study has demonstrated the increased in Npy expression stimulated food intake and hence resulted in weight gain [34]. Similarly, obese individual with non-alcoholic fatty liver diseases was found to have up-regulation of opioid signalling including Sigmar1 [35]. On the other hand, the expression level of Insr was up-regulated. Insr, the heterodimer genes coding for insulin signalling members, have been reported to be significantly reduced in obesity [36]. Thus, the up-regulation of Insr in the present study may lead to feedback down-regulation to reduce food intake due to increasing body weight of H-fed rats. The exact mechanism, however, is unknown.

There was report that bioactive compounds and nutrients in food can interact with the genome by highly complex forms [37]. For instance, administration of oleic acid through intracerebroventricular was found to decrease body weight by reduction of Npy and increase Pomc neuron [38]. Additionally, Lu et al. [15] found that supplementation of green tea polyphenols in high-fat induced obese rats reduced body weight through mediating obesity-related genes. In the present study, the expression of Insr and Pomc increased in obese rats supplemented with red pitaya while no changes were observed in seven genes (Adipor1, Apoa4, C3, Gcgr, IL-1r1, Insr, Npy, Pomc, Sigmar1 and Thrb). There was no statistical significance between the HRP group and H group that warrant future research with bigger sample size. Insulin plays critical roles in energy functions particularly in carbohydrate and lipid metabolism. Obese individual usually has marked declined in insulin levels [5]. Thus, the increased gene expression level of Insr in HRP group indicating that red pitaya ameliorates the deficiency or resistant of insulin in obese rats. Meanwhile, Pomc is important for central regulation of energy balance whereby it reduce food intake and increase energy expenditure via the release of α melanocyte-stimulating hormone (MSH) and activation of melanocortin receptors [39].

In addition, CRP group revealed the decreased expression of anorectic genes, Apoa4, Cntfr, Ghr and IL-1α. Among these, only IL-1α and Cntfr provides a significant reduction. As mentioned before, the increase expression of IL-1α and Cntfr induce satiety which in turn reduce the food intake and promote weight loss. Ghr plays a critical role in lipolysis, adipogenesis and lipogenesis and lower Ghr expression in the adipocyte is associated with obesity [40]. Likewise, Apoa4 triggered the satiety signal through dietary fat [41] and the decreased gene expression level of Apoa4 contributed to weight gain [15]. These findings confirmed that the lean control rats in the present study were more sensitive to the palatability of red pitaya juice as compared to high-carbohydrate, high-fat diet-induced obese rats. Red pitaya juice is an example of palatable foods that inhibit the satiety signals and hence increasing the food intake of CRP rats. Red pitaya juice contains higher amount of energy-supplying macronutrients causing progressive increment in body weight, and total body fat throughout the intervention period. This could explained the increased in abdominal fat deposition in CRP rats in the absence of a high carbohydrate, high fat diet.

As discussed earlier, the analysis on the obesity-related genes after supplementation with red pitaya juice resulted in the detection of 14 genes. The remaining 70 genes were not detected in the liver samples of all the three groups. The likely reasons for this condition are those undetected genes were weakly expressed or not expressed at all in the liver tissues. For instance, Calcr is typically expressed in the kidney and brain, Cartpt is normally expressed in the spinal cord, testis, prostate and brain while Brs3 is normally detected in the testis, kidney and brain [42]. Moreover, Lu et al. [15] suggested that the gene array may have limitation in the sensitivity of the detection. The authors also proposed that the changes in gene expression might not be detected in the mixture of cell types in tissue samples.

Interestingly, data showed that supplementation of red pitaya juice decreased the circulating C-reactive protein (CRP), thus reducing the diet-induced low grade inflammation *in-vivo*. A possible explanation for this might be that red pitaya supplementation reduced CRP concentration by reducing its production rate without altering the liver genes. Mauger et al. [43] reported that the main determinant for CRP concentration is its production rate which showed significant association with metabolic syndrome characteristics. In contrast, a recent study on the mechanism of CRP reduction by statins, a drug used for the treatment of hypercholesterolemia, indicated that its production was not reduced but the fractional catabolic rate was enhanced [44]. It is speculated that the increased in pro-inflammatory cytokines (IL-6 and TNF-alpha) was due to increase in liver fat as supplementation of red pitaya added to total energy content in high carbohydrate, high fat diet. Although the secretion of IL-6 regulates the induction of CRP in hepatocytes [45], the present study found contradicts results. As red pitaya supplementation increased IL-6, plasma concentration of CRP was reduced. In agreement with the present study, Malavazos et al. [46] found no positive association between IL-6 and CRP. In fact, a recent study reported that the intake of statin lowered CRP concentration but the study found no association between statin use and other inflammatory cytokines especially IL-6, TNF-α, and IL-1β [47].

## Conclusions

In summary, the present results demonstrated that genetic mechanisms play a major role in determining body weight by controlling energy balance homeostasis. The present study provides striking results in that the changes in anorectic, orexigenic and energy expenditure related genes not only to advance the understanding of beneficial effects of red pitaya, but the usefulness of this diet-induced rat model. However, the overall effects of red pitaya supplementation are still controversial as inconsistent results were obtained. Longer duration of study may be required. Furthermore, it is important to highlight the complexity of energy balance homeostasis that may be time-specific, and/or tissue- and species specific [48]. Nevertheless, the changes in anorectic, orexigenic and energy expenditure related genes observed in the present study provide the salient and novel findings for obesity research. This work can be further extended to investigate possible therapeutic effects of red pitaya supplementation on metabolic syndrome patient which will in turn help to fight against the obesity epidemic.

## Abbreviations

Actb, actin, beta; Adipor1, adiponectin receptor 1; Apoa4, apolipoprotein A-IV; Brs3, bombesin-like receptor 3; C3, complement component 3; Calcr, calcitonin receptor; Cartpt, CART prepropeptide; cDNA, complementary DNA; Cntfr, Ciliary neurotrophic factor receptor; CRP, corn starch + red pitaya juice; CRP, C-reactive protein; CS, corn starch diet; CT, threshold cycle; DNA, deoxyribonucleic acid; ELISA, enzyme-linked immunosorbent assays; Gcgr, glucagon receptor; gDNA, genomic deoxyribonucleic acid; Ghr, growth hormone receptor; HCHF, high carbohydrate, high fat; Hprt1, hypoxanthine phosphoribosyltransferase 1; HRP, high fat diet + red pitaya juice; IL-1, interleukin 1; IL-1r1, interleukin 1 receptor, type I; IL-1α, interleukin 1 alpha; IL-1β, interleukin 1 beta; IL-6, interleukin 6, Insr, insulin receptor; Ldha, lactate dehydrogenase A; mRNA, messenger ribonucleic acid; MSH, melanocyte-stimulating hormone; NPY, neuropeptide Y; PCR, polymerase chain reaction; Pomc, proopiomelanocortin; RNA, ribonucleic acid;Rpl13a, ribosomal protein L13A; Rplp1, ribosomal protein, large, P1; RT, reverse transcription; SEM, standard error of mean; Sigmar1, sigma non-opioid intracellular receptor 1; Thrb, thyroid hormone receptor beta; Tnf, tumor necrosis factor
